# TMB-Hunt: An amino acid composition based method to screen proteomes for beta-barrel transmembrane proteins

**DOI:** 10.1186/1471-2105-6-56

**Published:** 2005-03-15

**Authors:** Andrew G Garrow, Alison Agnew, David R Westhead

**Affiliations:** 1 School of Biochemistry and Microbiology, University of Leeds, Leeds, LS2 9JT, UK

## Abstract

**Background:**

Beta-barrel transmembrane (bbtm) proteins are a functionally important and diverse group of proteins expressed in the outer membranes of bacteria (both gram negative and acid fast gram positive), mitochondria and chloroplasts. Despite recent publications describing reasonable levels of accuracy for discriminating between bbtm proteins and other proteins, screening of entire genomes remains troublesome as these molecules only constitute a small fraction of the sequences screened. Therefore, novel methods are still required capable of detecting new families of bbtm protein in diverse genomes.

**Results:**

We present TMB-Hunt, a program that uses a *k*-Nearest Neighbour (*k*-NN) algorithm to discriminate between bbtm and non-bbtm proteins on the basis of their amino acid composition. By including differentially weighted amino acids, evolutionary information and by calibrating the scoring, an accuracy of 92.5% was achieved, with 91% sensitivity and 93.8% positive predictive value (PPV), using a rigorous cross-validation procedure.

A major advantage of this approach is that because it does not rely on beta-strand detection, it does not require resolved structures and thus larger, more representative, training sets could be used. It is therefore believed that this approach will be invaluable in complementing other, physicochemical and homology based methods. This was demonstrated by the correct reassignment of a number of proteins which other predictors failed to classify. We have used the algorithm to screen several genomes and have discussed our findings.

**Conclusion:**

TMB-Hunt achieves a prediction accuracy level better than other approaches published to date. Results were significantly enhanced by use of evolutionary information and a system for calibrating *k*-NN scoring. Because the program uses a distinct approach to that of other discriminators and thus suffers different liabilities, we believe it will make a significant contribution to the development of a consensus approach for bbtm protein detection.

## Background

### Beta-barrel transmembrane proteins

The beta-barrel is one of only two membrane spanning structural motifs currently identified [1]. It is proven with high resolution structures for many proteins expressed within the outer membranes of gram negative bacteria and is also widely expected for several proteins expressed in the outer membranes of mitochondria [2] and chloroplasts [3]. In addition, the structure of a protein found spanning the outer membrane of *Mycobacteria *(an acid fast gram positive bacterium) was recently resolved revealing two consecutive membrane spanning beta-barrels [4]. As with alpha-helical transmembrane (ahtm) proteins, beta-barrel transmembrane (bbtm) proteins play both functionally important and diverse roles [1].

Currently, over 92 bbtm protein structures are present in the protein databank [5], including 23 families as defined in PDB_TM [6]. They are classified in the SCOP hierarchy, in 3 different folds [7], the transmembrane beta-barrels (described as not a true fold, but a gathering of beta-barrel membrane proteins), the integral outer membrane protein TolC fold and the Leukocidin (pore forming toxins) fold. The transmembrane beta-barrels consist of four SCOP superfamilies; OmpA-like, OmpT-like, OmpLA and the Porins; and include channels, enzymes and receptors. These superfamilies vary in numbers of subunits, where each subunit contributes a single barrel. The TolC fold, consists of one SCOP superfamily and includes proteins involved in secretion and expression of outer membrane proteins (OMPs) [8]. These proteins are trimeric with each subunit contributing four strands to a single barrel, and contain large stretches of alpha-helix, which stretch across the periplasm. Finally, the Leukocidin fold consists of heptameric pore forming toxins with each subunit contributing 2 strands to the barrel. TolC, Leukocidin and the *Mycobacterial *porin MspA (which is not yet classified within SCOP) can thus be considered "non-typical" bbtm proteins. From the diversity of bbtm proteins in different SCOP folds, it seems likely that these proteins have multiple evolutionary origins.

These structures have helped reveal a number of features concerning transmembrane (TM) beta-strands and their organisation [9]. TM beta-strands show an inside-outside dyad repeat motif of alternating residues facing the lipid bilayer and the inside of the barrel. Outside (lipid bilayer facing) residues are typically hydrophobic whilst inside (facing inside of barrel) residues are of intermediate polarity. TM beta-strands are often flanked by a layer of aromatic residues, believed to be involved in maintaining the protein's stability within the membrane [10]. Structures have also revealed an even number of strands, with N and C termini on the same side of the membrane. Strands form an antiparallel beta-meander topology with alternating long and short loops. The number of TM beta-strands in a barrel has been shown to range from 8–22 strands, with a range of 6–22 (most frequently 12) residues.

In contrast to ahtm proteins, which are easy to identify through TM alpha-helices composed of 20 or more hydrophobic residues [11], the short and cryptic nature of TM beta-strands makes their discrimination difficult. Prediction is complicated further with beta-strands of some globular proteins superficially resembling those of bbtm proteins.

### BBTM protein discriminators

Despite these difficulties, numerous methods have recently been published for the identification of these proteins, most commonly focusing on identification of TM beta-strands. Methods include rule based approaches [12], an architecture based approach [13], Hidden Markov Models (HMMs) [14-18], a neural network based method [19], a combined neural network and support vector machine [20], composition of transmembrane beta strands combined with secondary structure prediction [21] and an approach based on architecture [13] combined with isoleucine and asparagine abundance [22]. Of these, the first two give no indication of discriminatory accuracy, but the others range from 80 to 90%.

Whilst this level of accuracy may seem acceptable if analysing a particular sequence of interest, problems will occur when screening an entire genome for potential bbtm proteins, owing to the fact that a large number of sequences are being tested of which these molecules only constitute a small fraction. There is therefore a need for programs with higher accuracy and in particular higher specificity, in order to minimise the false discovery rate.

### Amino acid composition based protein classification

This paper describes TMB-Hunt, an amino acid composition based program for the identification of bbtm proteins. Amino acid composition has been analysed for bbtm proteins [13], however whole sequence composition has not previously been used for discrimination. Many previous studies have shown how amino acid composition can be successfully applied to protein sequence analysis, including prediction of structural class [23], discrimination of intra- and extra cellular proteins [24] and distinguishing between membrane protein type [25]. Amino acid composition is often used for prediction of subcellular location, as an alternative to signal detection based methods [26-29] which are prone to errors in automated gene prediction at the 5' end [30]. The limitation of this technique, however, is that the correlation of cell location with amino acid composition is not absolute. It was suggested that composition differences are a consequence of different requirements for protein folding, stability and transportation [24,26]. Subsequently it has been shown that amino acid composition differences correlate most strongly with surface residues [27]. Thus, composition has been particularly useful in discriminating between ntm and ahtm proteins, which consist of large numbers of hydrophobic amino acids in contact with the lipid bilayer. This feature has enabled algorithms to be developed capable of distinguishing between the two classes with >97% accuracy [31], based on identification of the TM alpha-helices.

Because TMB-Hunt puts no emphasis on identification of TM beta-strands, we were not dependent on sequences with resolved structures and training sets could be much larger than those used for other predictors [12-22]. As a result, bbtm proteins with structures more diverse than those used by other predictors were included, resulting in a greater degree of sensitivity. TMB-Hunt is at least as accurate as other predictors, but its major advantage is that it adopts a completely different approach to other methods and is likely therefore to be valuable in consensus approaches, which should be much more successful at hunting for new families of candidate bbtm proteins in diverse proteomes.

## Implementation

### Training sets

Training sets for bbtm, ahtm and non-TM (ntm) proteins were gathered from a number of manually curated and published sources. The PDB accessions of 3159 ntm proteins were acquired from PDB-REPRDB via the Papia database [32], and respective sequences were extracted.

Sequences of ahtm proteins were downloaded from a test set available at the Sanger centre [33]. Four datasets were available of varying quality. Dataset A comprised 37 sequences where structural information was available. Dataset B contained 23 sequences with very good biochemical characterisation from at least two complementary methods. Dataset C contained 129 sequences with some biochemical characterisation and where annotation was only reliable for part of the sequence. Dataset D contained sequences with no biochemical characterisation and only hydrophobicity or an alignment as a basis for their characterisation. Datasets A, B and C were used.

Beta-barrel transmembrane protein sequences were downloaded from a number of resources including:

957 from UniProt [34] using a keyword search for 'Transmembrane' and 'Outer Membrane' and taxonomy filter for only bacteria

134 from the transporter classification (TC) database [35]

35 extracted from the PDB files of beta-barrel outer membrane proteins in SCOP [7].

All these datasets were manually created and rechecked to ensure no obvious spurious sequences were present. Sequences of less than 120 residues were removed from the training set. Sequences were next grouped into clusters using BLASTclust and a sequence similarity threshold of 23%. Amino acid composition profiles were produced for each group using evolutionary information, as described below. Dataset details are summarised in Table 1.

The final dataset included numerous types of bbtm protein not included in the training sets of other predictors. Inclusion of such a diverse range of proteins was important as it covers a wide range of evolutionary origins and physicochemical adaptations. TolC, Alpha-hemolysin and the Mycobacterial Porin Family are bbtm proteins with resolved structures, not used by other predictors, either because of their unusual structure or because their structure was resolved after the predictor had been completed. Fimbrial, pili and flagellar associated proteins were also included, as were non-bacterial proteins e.g. the mitochondrial porin (VDAC), plastid bbtm proteins (e.g. OEP24) and chloroplast porins (Toc75).

Sequences used for proteome screening were downloaded from the NCBI FTP site [36]. Sequences used for annotation comparison were downloaded via SRS [37,38] from Uniprot [34].

### *k*-nearest neighbour algorithm

The *k*-nearest neighbour algorithm is a simple instance-based learning method for performing general, non-parametric classification [39,40]. Each object or instance (a protein in this case) is associated with a class which can be unknown (class 0), bbtm (1), ahtm (2) or ntm (3). For query proteins of unknown class, predictions are made by using information from a training set of proteins where the class is known. The prediction is made on the basis of a set of *k *objects from the training set which are most similar (in the sense described below) to the query protein. This technique is thus a local approximation, focusing on the neighbourhood of the query instance. A major advantage of this algorithm is that it is robust to noisy data (given a large dataset), as taking the weighted average of the nearest neighbours smoothes out isolated training instances.

Proteins are represented by *x *= (*f*_*a *_(*x*), *a *∈ A; *c*(*x*)), where *c*(*x*) represents the class *c *∈ {0,1,2,3} as defined above, A is the set of naturally occurring amino acids and *f*_*a*_(*x*) denotes the relative frequency of the amino acid *a*. The distance between two proteins *x*_*i *_and *x*_*j *_in this representation is measured by the standard Euclidean metric.



Given a query protein *x*_*q*_, the algorithm first finds the *k *closest instances in the training set according to this metric, and then assigns a score S(*x*_*q*_, *c*) for each possible class *c*,



where *δ*(*c*_1_, *c*_2_) = 1 if the classes *c*_1 _and *c*_2 _are equal and zero otherwise. Thus the score for each class is a sum of positive contributions from each of the nearest neighbours from that class, where the contribution is weighted according to the reciprocal square distance between query instance and neighbour with closer neighbours contributing more strongly.

Since we are very often concerned with binary classification problems (e.g. distinguishing bbtm proteins from proteins in any other class), it is also useful to define a discrimination score,



which is the score from one class (e.g. bbtm proteins) minus the scores from other classes.

### Calibration and scoring

In making predictions a standard nearest neighbour algorithm would simply predict the class of *x*_*q *_to be the class *c *with the highest score *S*(*x*_*q*_, *c*). However, this procedure is problematical in cases such as this where the training set is unbalanced, containing many more ntm proteins than either of the other two classes. Statistical chance means that the *k*-nearest neighbour sets tend to contain more proteins from the dominant class, leading to this class as the dominant prediction even in the presence of substantial evidence for membership of one the other classes in the nearest neighbour set. One approach to this problem would be to reduce representation of the dominant class to produce a balanced training set, but this procedure involves wasting useful information. It would also be possible to down-weight information from the dominant class, but we found that a more effective approach was to use the distributions of *D*(*x*,*c*) scores in the training set proteins, divided between proteins in class *c*, and proteins in other classes from which they are to be distinguished. For clarity, in the remainder of this section we will consider *c *= 1, where the classification problem is to distinguish bbtm proteins from any others, and *D *will denote the discrimination score *D*(*x*,*c *= 1) for an arbitrary protein *x*.

**Figure 1:**
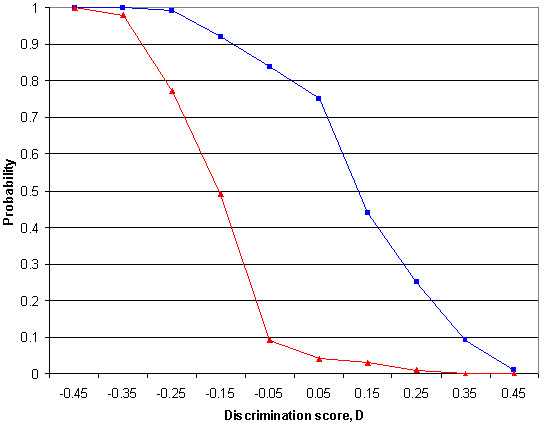
**Probabilities used for development of a calibrated score. **Probability (y-axis), p(D'≥D), for observing a score D' greater than or equal to D (x-axis) for either bbtm (■) or ntm (▲) proteins. Plots were made by calculating the frequencies of bbtm and ntm proteins identified above certain discrimination scores (using weighted amino acids, no evolutionary information and a 'leave homologues out' cross-validation).

Empirical cumulative probability distributions for *D *in the case above are shown in Figure 1. As expected, plots showed a higher mean discrimination score for bbtm (mean = 0.078, standard deviation = 0.115) than other proteins (mean = -0.206, standard deviation = 0.171). These distributions do not deviate significantly from the normal distribution. Using these distributions it is possible to convert discrimination scores into a convenient log likelihood ratio (beta-barrel score),

*R*(D) = log(*p*(*bbtm*|*D*)/*p*(*other*|*D*)),

where *p*(*bbtm*|*D*) denotes the probability of a bbtm protein obtaining a score of *at least D*, and *p*(*other*|*D*) denotes the probability of a protein from the other class obtaining a score of *D or greater*. Negative values of *R *indicate a query protein more likely to come from the other class, and positive values indicate a protein more likely to come from the bbtm class.

An alternative probabilistic interpretation of the *D *score is the expected number of proteins from the other class scoring *D *or greater, *E*(*D*) = *Np*(*other*|*D*), where N indicates the number of query sequences tested. This measure takes account of the multiple testing involved in screening large numbers of sequences in a genome, and is related to the standard Bonferroni correction. It is directly analogous to the E-values reported by the popular sequence search programs FASTA [41] and BLAST [42].

### Differential dimension weightings

To account for some dimensions contributing information more valuable to classification than others, weights were applied to each of the dimensions used in calculating Euclidean distances. The modified Euclidean distance calculation was:



where *g*_*a *_is the weight applied to amino acid *a*.

A genetic algorithm was employed to calculate the optimal weightings for each dimension. Genetic algorithms are an optimisation approach, based on Darwinian principles, which assume that given a population of individuals, environmental pressures cause natural selection thus increasing the overall fitness of the population [43]. Application of a genetic algorithm requires a population of solutions, termed chromosomes, whose fitness can be measured using an objective function. Based on fitness, the better candidates are chosen to seed the next generation through a combination of crossover and/or mutation. This will result in the evolution of successively better solutions. The process is carried out until an optimal solution or time limit is reached.

The algorithm initiates by constructing a random population of chromosomes (i.e. potential solutions), represented as vectors, with each element of the vector termed a gene, representing a weight for a particular dimension of the Euclidean space. Fitness for chromosomes was measured by the Matthews Correlation Coefficient (MCC) value returned from a 'leave homologues out' cross-validation analysis (see below) using a fixed set of 100 bbtm proteins and 100 ntm proteins. Once fitness for each of the chromosomes within a generation was determined, the fittest were used to create offspring through a process of crossover and mutation. Crossovers involve the construction of a new vector, using random genes taken from two or more parents. Mutations involved randomly mutating 1 in 8 genes.

### Inclusion of evolutionary information

Random noise in amino acid composition was reduced by inclusion of evolutionary information. Evolutionary information was included by building a feature vector using both the query sequence, as well as a number of close homologues (as determined by a BLAST query against Uniprot/SwissProt with an E-value threshold of 0.0001, and a maximum of 25 homologues) to calculate an average amino acid composition vector for the sequence and its close evolutionary relatives. A weighted average composition was used, with more distant homologues contributing more to the average (since the more distant sequences contain more new information). Weights were assigned by first carrying out all-against-all alignments within the set using BLAST, then weighting sequences according to their average distance to other sequences. The weights were calculated as



where *W*_*k *_denotes the weight applied to sequence *k*, and *p*_*k *_the average percentage difference (100 minus the percentage identity) from sequence *k *to other sequences.

### Performance

Cross-validation studies were used to assess performance. Two approaches were used, 'leave-one out' cross-validations and 'leave-homologues out' cross-validations. The first of these methods involved removing in turn profiles from the training set and seeing if the algorithm could correctly reassign one of the sequences used to build the profile. Removal of profiles and their construction using sequences in clusters of >23% identity meant that sequences should not then be correctly reassigned due to 'self-detection' by a close homolog. However, even sequences of <23% identity can be homologues and show significant similarity e.g. over shorter fragments of the sequence, therefore a 'leave homologues out' cross-validation was used as a stricter alternative. This meant pre-computing sequences similar (with a BLAST E-value threshold <1) to each query sequence, and leaving these out of the training set when testing. This procedure eliminates any homolog whose sequence is sufficiently similar to be detected with BLAST.

Performance was measured using sensitivity, specificity, positive predictive value (PPV), negative predictive value (NPV), accuracy and MCC, which are defined in terms of true positives (TP), false positives (FP), true negatives (TN) and false negatives (FN).

Sensitivity is a measure of the percentage of bbtm proteins correctly classified and is calculated with, 100*TP/(TP+FN). Specificity is the percentage of non-bbtm correctly classified as is calculated as 100*TN/(TN+FP). The PPV is the percentage of predicted bbtm proteins that are correct and is calculated by, 100*TP/(TP+FP). The NPV is the percentage of predicted non-bbtm proteins that are correct and is calculated using 100*TN/(TN+FN). Accuracy is a measure of the total number of correctly assigned proteins and is measured by, 100*(TP+TN)/*t*, where *t *is the total number of sequences queried. However this statistic can be misleading in circumstances with bias in the test set composition. Therefore, the Matthews Coefficient Correlation (MCC) is an alternative measure that accounts for both under and over predictions.



This returns a value between -1 and 1, with 1 meaning everything is correctly assigned and -1 meaning everything is incorrectly assigned. Given two prediction classes (e.g. bbtm and ntm) and a random probability of assigning queries to either, a score of 0 would be expected by random classification.

## Results

TMB-Hunt uses a *k*-Nearest Neighbour (*k*-NN) algorithm to classify query instances, using the class (bbtm, ahtm or ntm) of their nearest neighbours, as defined by differences in amino acid composition. A number of steps were involved in optimisation, including selection of the numbers of neighbours used (*k*), amino acid weightings and scoring statistics. Once optimised, performance of the program was assessed and it was applied to the screening of several genomes.

### *K*-values

An optimal *k*-value was chosen using a series of cross-validation tests. These were computed with a range of parameters and, consistently, the program found that accuracy showed a weak peak at *k *= 5 and gradually declined thereafter. However performance was generally insensitive to the precise value of *k*, with similar performance shown for moderate values ≥ 5.

### Differential amino acid weightings

**Figure 2:**
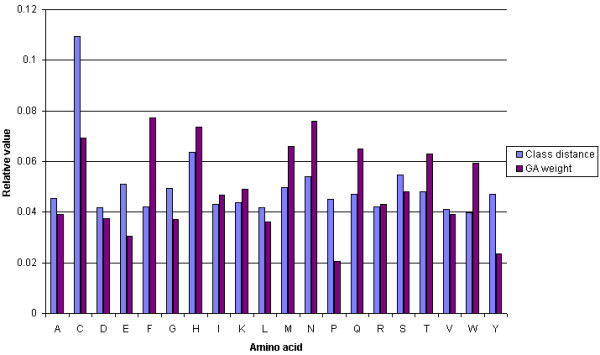
**Comparison between GA weightings and difference ratios. **Relationship between GA derived weights for amino acids and weights based simply on average compositional distances between classes.

A genetic algorithm was used to calculate optimal amino acid weightings for differentiating between bbtm and ntm proteins. The results are shown in Figure 2, alongside weights derived from average compositional differences between the classes. Amino acids contributing the most to classification include Cys, Phe, His, Met, Asn, Gln and Thr. Those contributing the least include Glu, Pro and Tyr. The greatest contributing amino acid, Phe contributed 3.76 times more than the lowest, Pro.

Interestingly, these weights did not completely correlate with compositional differences (Figure 2). Phe had the greatest GA weighting, with 0.077, but had a relatively small composition difference between training sets, with corresponding weight 0.042 (ranked 15^th ^of 20) and Glu had a fairly large composition difference (ranked 7th) but lower GA weighting (ranked 16th). However, there were some correlations, with Asn, His, Cys and Met ranked 2^nd^, 3^rd^, 4^th ^and 5^th ^in the GA weightings and 4^th^, 2^nd^, 1^st ^and 6^th ^respectively in the composition difference rankings.

Weights significantly differed from those used by Liu [21] who found, using a Fisher's Discrimination Ratio, that the amino acids most useful for distinguishing between beta-strands of globular and membrane proteins were Gly, Val, Ile, Asn, Leu and Cys. These differences can be attributed to the fact that Liu tried to identify differences in strand residues, whereas our method identifies differences in the composition of entire sequences.

### Performance

**Figure 3:**
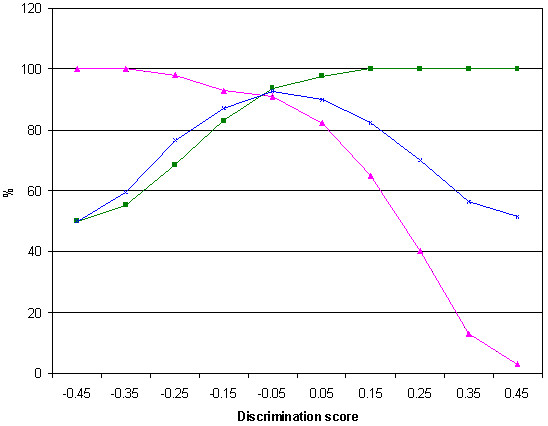
**TMB-Hunt performance over a range of discrimination scores. **Accuracy (x), sensitivity (▲) and PPV(■) of the predictor at range of discrimination score thresholds. The above results were taken for the predictor discriminating between bbtm and non-bbtm proteins, using the 'leave homologues out' cross-validation, with weighted amino acids and evolutionary information for the query sequence. Similar patterns were found with all settings i.e. using weighted amino acids, no evolutionary information, 'leave homologues out' cross-validation and discriminating between bbtm and ntm proteins.

The ability of the program to discriminate between different classes was tested using a 'leave homologues out' cross-validation (see methods) and was defined in terms of PPV, sensitivity and accuracy. Figure 3 shows how PPV, sensitivity and accuracy vary over a range of discrimination scores. Performance results are summarised in Tables 2,3, with the optimal cut-off point (discrimination score giving the highest accuracy) used. Table 2 summarises the performance difference between the program with various features, i.e. weighted amino acids and query sequence evolutionary information. Table 3 describes the ability of the program to discriminate between various protein classes with two different settings. Without inclusion of query sequence evolutionary information, the program was better at discriminating between bbtm and ntm proteins than bbtm and ahtm, with accuracies of 85% and 77.5% respectively. This difference was reduced with the inclusion of query sequence evolutionary information and weighted amino acids, with a prediction accuracy of 92.5% for discrimination between both bbtm and ntm proteins and bbtm and ahtm proteins.

Results reported so far have used cross-validations based on removing detectable homologues (BLAST E-value<1) from the training set. The results have shown high accuracy discriminations. This indicates that amino acid composition can be used to identify bbtm proteins. It is not possible to know the extent of very distant homology in the training set, since this is often only apparent when 3D structures are determined. It is not clear therefore whether the good performance we observe results from the detection of distant homologues, or whether the composition signal is a characteristic of many evolutionary unrelated families of bbtm protein. It seems likely that both explanations contribute to the results, which indicate at the very least that composition is an important feature of these proteins that is preserved over long evolutionary distances and may be shared by unrelated bbtm proteins.

The program was extremely fast, able to query 400 sequences in <1 minute on a 2 Ghz Pentium processor. When using evolutionary information, speed was limited by a BLAST query against Uniprot/Swissprot, and 'all against all' BLAST runs to identify the similarities of homologues. However, even with evolutionary information TMB-Hunt is still faster than Prof-TMB, of a similar speed to Pred-TMBB and only marginally slower than BOMP.

### Specific examples

Cross-validation results were reviewed specifically for a number of bbtm proteins that are non-typical, controversial, expressed in membranes other than the outer membrane of gram negative bacteria or for bbtm proteins of gram negative bacteria that have recently been structurally resolved. The aim of TMB-Hunt is identification of novel families of bbtm protein. Unfortunately a fair comparison of the abilities of various predictors to detect novel families is difficult owing to unavoidable uncertainties about training set contents and in some cases (e.g. BOMP) a lack of user control in specificity thresholds. In an attempt to make this comparison we chose examples that for the reasons given should not be well represented in the training sets of other predictors. The ability of TMB-Hunt to identify novel families is given with results coming from cross-validation tests. Table 4 gives details of prediction results using TMB-Hunt and compares them with three other web-based bbtm protein predictors; BOMP, Prof-TMB, Pred-TMBB.

Pred-TMBB and TMB-Hunt both correctly classified non-typical bbtm proteins TolC [8] (P02930), Alpha-hemolysin [44] (P09616) and the Mycobacterial Porin [4] (Q9RLP7), whilst these were classified as non-bbtm by BOMP and Prof-TMB. The secreted pore-forming toxin, Alpha-hemolysin is difficult to classify because the majority of its beta-strands are non-membrane. Alpha-hemolysin is homoheptameric, with each subunit contributing 2 strands to a 14 strand TM barrel. In addition to the 2 TM strands, each subunit consists of 14 soluble strands which make up a cap and rim domain. The Mycobacterial Porin, has not been included in the training sets of any currently published predictors, because its structure has only recently been resolved [4] and because, at 10 nm width, the outer membrane of gram positive Mycobacteria is unlike that of gram negative bacteria at 4 nm width [45]. TolC has been a problem in classification because each of the three subunits contributes just 4 strands to the beta-barrel and contains large stretches of alpha-helix.

To confirm that the predictor was not just selecting proteins destined for the outer membranes of gram negative bacteria, we also tested with a number of mitochondrial and chloroplast bbtm proteins. All the predictors tested were able to correctly classify the mitochondrial porin VDAC (Q9RLP7), but only BOMP and Pred-TMBB classified Tom40 (Q18090) as a bbtm protein. Only Prof-TMB and TMB-Hunt (using the 'leave-one out' cross-validation) classified Toc75 (Q43715) as a bbtm protein and only Pred-TMBB and TMB-Hunt identified OEP24 (O49929).

All four predictors tested were able to correctly identify proteins with recently resolved structures i.e. Tsx [46] (P22786), FadL [47] (P10384), BtuB [48] (P06129) except BOMP which misclassified NalP [49] (Q8GKS5). BOMP was the only predictor tested which did not classify Secretin [50] (P31700) as a bbtm protein but all four classified the Usher protein [51] (P30130) as bbtm. A 60 kDa cysteine rich outer-membrane protein [52] (P26758), was the only example that was not classified as a bbtm protein by any of the predictors. However the experimental evidence that this is a genuine bbtm protein is weak and it has been suggested that it is falsely annotated [21]. It should be noted that PSORT-B 2.0 [53] identified all of these examples as outer membrane proteins, including the 60 kDa rich outer membrane protein. However it classified these using strong homology to sequences within its training set and thus did not give a representation of its ability to predict novel families of bbtm proteins.

Differences in the prediction results of these algorithms with these examples suggests that combined approaches could result in a higher overall accuracy.

### Genome screening

**Figure 4:**
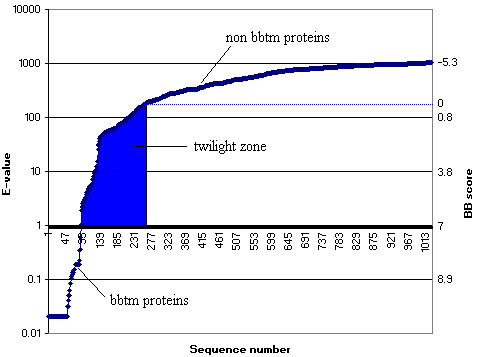
**Range of E-values and BB-scores from *E. coli *screening. **Sequences with a predicted signal peptide from the proteome of *E. coli*, were screened using the algorithm described. Sequences were then sorted by their E-values and plotted graphically. The graph demonstrates that in proteome screening with this tool there a number of sequences will be identified with positive bb scores, but E-values >1. Sequences with these scores are described as being in the twilight zone.

Figure 4 demonstrates typical results seen when screening a genome. It demonstrates that due to the large number of sequences queried, a number of sequences get scores with an E-value >1 but a beta barrel score indicative of a bbtm protein (i.e. >0). These sequences are said to be in the 'twilight zone' because it is impossible to classify them as either bbtm or not. To reduce the number of sequences within this zone, sequences without signal peptides were removed. Sequences were accepted if a signal peptide was predicted using SignalP 3.0 with either the Neural Network [54] or HMM [55] modes, so as to minimise the number of potential candidates removed. Similar filtering systems have been applied in previous bbtm protein screening attempts [3,16,56]. Signal peptide filtering poses certain risks owing to errors in the prediction of the 5' ends of genes [30] and imperfections in signal peptide prediction algorithms, but these risks are outweighed by the reduction of FP sequences within the twilight zone.

A range of organisms with completed genomes were screened for bbtm proteins, including several bacteria, a protozoan, a fungus, a nematode and an angiosperm. Table 5 shows the results of proteomes screened. *Plasmodium falciparum, Saccharomyces cerevisiae*, *Caenorhabditis elegans *and *Arabidopsis thaliana *were screened as eukaryotic tests. To date, the only predicted eukaryotic bbtm proteins are those of the mitochondrial and chloroplast outer membranes, however the possibility of other eukaryotic bbtm protein families should not be ignored. Three examples of where they could exist are i) organelles of endosymbiotic bacterial origin other than the mitochondria and chloroplasts e.g. the apicoplast of apicomplexan parasites including the malaria parasite *Plasmodium *[57] or ii) novel double membrane systems e.g. the outer membranes of the parasitic worm schistosomes, which contains two overlaid phospholipid bilayers [58] and iii) toxins e.g. TT95 which is a pore forming molecule produced by the parasitic nematode *Trichuris *[59] but which does not contain any predicted TM helices.

Screening eukarotic genomes for bbtm proteins is a more complex process than with prokaryotes owing to larger numbers of sequences queried and a wider range of targeting signals. TMB-Hunt is able to identify mitochondrial and chloroplast outer membrane bbtm proteins (Table 4), but these were missed during eukaryotic genome screening due to prior removal of sequences without signal peptides. Owing to the wide range of eukaryotic protein targeting pathways, eukaryotic sequences should ideally be screened without prior filtering, however this would result in much larger numbers of sequences within the twilight zone. Another alternative would be an addition to the score whenever targeting signals are detected.

TMB-Hunt did not predict many bbtm proteins in eukaryotes; 3 with an E-value <1 in *P. falciparum *(0.03% of all proteins screened), 4 in *S. cerevisiae *(0.07%), 23 in *Arabidopsis thaliana *(0.07%) and 26 in *C. elegans *(0.1%), with the majority of selected sequences in *A. thaliana *and *C. elegans *being closely related and described as hypothetical or putative proteins. Only 1 eukaryotic protein got an E-value <0.1, a *P. falciparum *gene annotated as a serine protease with an E-value of 0.032.

The mean percentage of proteins in Gram negative bacterial proteomes, with an E-value <1, was 1.37%, with a range of 0.65–2.46%. The figure was highest in proteobacteria, possibly reflecting biases in the training set, with homologies to training instances enabling statistically significant scores (E-values) for many sequences. However given that the numbers of bbtm proteins in various bacterial phyla is not known, it may be that these results reflect true figures. Previous results [17] identified smaller numbers of bbtm proteins in some genomes e.g. *Aquifex aeolicus*, *Thermatoga maritima *and *Trepanoma palidium *although the numbers of sequences screened were not given.

**Figure 5:**
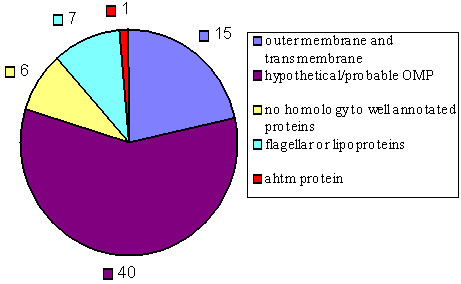
**Uniprot annotation of predicted *E. coli *bbtm proteins. **Numbers of *E. coli *O157:H7 sequences with a TMB-Hunt E value <= 1 with different categories of annotation in Uniprot.

*Escherichia coli O157:H7 *proteins downloaded from Uniprot were screened in order to compare results with high quality annotation (Figure 5). In total, 249 sequences got a positive beta barrel score when, given the number of sequences queried, 133 would be expected. Thus assuming the remaining 116 sequences are genuine bbtm proteins, the proteome contains (116/4005) × 100 = 2.896% bbtm proteins (a number consistent with other predictions). Of these 249 sequences, 69 had an E-value<1, that is 1.72% of all proteins queried. These 69 included 15 proteins described as outer membrane and TM, 40 hypothetical or putative bbtm proteins described as probable OMPs or with homology to OMPs, 6 hypothetical proteins without homology to well annotated proteins, 4 flagellar proteins, 3 lipoproteins and 1 well known ahtm protein. The 15 proteins described as outer membrane and TM should be bbtm proteins and the 40 with homology to OMPs are probably bbtm proteins. The flagellar are possible bbtm proteins as several flagellar proteins are known bbtm proteins. The 6 hypothetical proteins without homology to well annotated proteins possibly represent novel families of bbtm protein. The 3 lipoproteins are non-bbtm proteins and the 1 ahtm protein could be easily filtered using a ahtm protein predictor.

TMB-Hunt proved successful in that Uniprot annotation suggests that the vast majority of bbtm proteins (65 of the 69 (>95%)) it predicted were probably bbtm proteins. However, several more probable bbtm proteins were found in the twilight zone, suggesting that this algorithm alone does not infallibly detect all bbtm proteins, even in organisms well represented in the training set. In comparing results with BOMP, we found it rejected the lipoproteins that TMB-Hunt incorrectly classified as bbtm (Q8XBQ1, Q7ABP6, Q7ABA4), whilst correctly classifying a number of proteins annotated as bbtm proteins which were within the TMB-Hunt twilight zone (e.g. Q7AGG6, Q7AY93). However we found that BOMP also incorrectly rejected a large number of annotated bbtm proteins that we classified with an E-value <1 (e.g. Q7AAR4, Q7A9N7). Similar patterns were found with Pred-TMBB and Prof-TMB. These differences are further evidence suggesting that combining algorithms could lead to a higher overall accuracy.

Because composition is correlated with physicochemical environment [26], TMB-Hunt struggles with differentiation between bbtm proteins and proteins occupying similar environments i.e. lipoproteins and periplasmic proteins. However TMB-Hunt gets a stronger signal from bbtm proteins as they effectively occupy 3 environments, the transmembrane (where there is a preference for amino acids which form TM beta-strands) and either side of it, whereas lipoproteins and periplasmic proteins will occupy only one side of the membrane. The liability of TMB-Hunt is thus different to that of topology based predictors which typically report difficulties in discriminating between beta-strands of bbtm proteins and some globular proteins.

## Conclusion

A program called TMB-Hunt has been described which identifies bbtm proteins using the amino acid composition of entire sequences. TMB-Hunt uses a novel method for calibration of results from the *k*-NN algorithm and uses evolutionary information from close homologues to build composition profiles. We suggest that these methods can be used to boost the accuracy of other *k*-NN and composition based classifiers.

TMB-Hunt was found to have several advantages over existing methods. Firstly, a cross-validation analysis showed performance to be superior to that of other bbtm protein predictors. Secondly, unlike previous predictors which are dependent on TM beta-strand detection, this method does not require resolved structures and thus larger more representative training sets could be used. Thirdly, by adopting a novel approach, we believe that the major benefit of this program is that it has different liabilities to others. This was demonstrated by its ability to correctly classify several proteins with which previous predictors struggled. Finally, it is extremely quick, capable of screening >400 sequences per minute. TMB-Hunt has been successfully applied to the screening of several genomes, however, numerous proteins fell into the twilight zone, where it was impossible to statistically categorise them as either bbtm or not. It is therefore intended that it will be included as part of a consensus approach, which can be used to hunt for novel families of bbtm protein.

## Availability and requirements

**Project name: **TMB-Hunt

**Project home page: **A web server is available at .

**Operating system: **LINUX

**Programming languages: **ANSI C and Perl

**Other requirements: **None

**Licence: **GPL

**Any restrictions to non-academics: **None

## Abbreviations

AA – Amino acid

ahtm – Alpha-helical transmembrane

bbtm – Beta-barrel transmembrane

BLAST – Basic Local Alignment Search Tool

GA – Genetic Algorithm

HMM – Hidden Markov Models

*k*-NN – *k*-Nearest Neighbour

MCC – Matthews Correlation Coefficient

ntm – Non Transmembrane

OMP – Outer membrane protein

PDB – Protein DataBank

PPV – Positive predictive value

TM – Transmembrane

## Authors' contributions

AGG constructed the datasets, wrote and tested the programs, screened genomes and built the website. AA suggested the project and analyzed the genome screening results. DRW oversaw the construction of the programs and helped develop the methods. All authors have read and approved the final manuscript.

## Supplementary Material

Additional File 1 TMB-Hunt source code and training sets Click here for file

Additional File 2 E. coli O157:H7 (Uniprot sequence) query results. Various proteomes screened, examples of results and queries, help files. Click here for file

## Figures and Tables

**Table 1:** Sequence datasets used to generate training sets.

**Training dataset**	**Sources**	**Initial number sequences**	**Sequences >120 AA**	**Size after redundancy removal**
ntm	PDB-REPRDB [32]	3159	2290	1763
ahtm	Sanger all-alpha membrane datasets A, B and C [33]	189	166	132
bbtm	TC-DB [35], Uniprot [34] and PDB [5]	1126	1107	196

Three training datasets were generated using sequences from various sources. Datasets were filtered for sequences of <120 AA and clustered to remove redundancy.

**Table 2:** Program performance using different settings.

***BBTM vs NTM***	**% Sensitivity**	**% Specificity**	**% PPV**	**% NPV**	**% Accuracy**
**Plain**	83	87	86.5	83.7	85
**Weighted AAs**	84	91	90.3	85	87.5
**Evolutionary information**	89	94	93.7	89.5	91.5
**Evolutionary information + weighted AAs**	91	94	93.8	91.3	92.5

Ability of the program to discriminate between bbtm and ntm proteins, using the 'leave homologues out' cross-validation method and with a range of different features. The plain mode indicates neither evolutionary information or weighted amino acids were included.

**Table 3:** Ability of program to differentiate between various protein classes.

***A. Plain***	**% Sensitivity**	**% Specificity**	**% PPV**	**% NPV**	**% Accuracy**
***bbtm vs ntm***	83	87	86.5	83.7	85
**bbtm vs ahtm**	83	72	74.8	80.1	77.5
					
***B. Evolutionary Information plus weighted AAs***	**% Sensitivity**	**% Specificity**	**% PPV**	**%NPV**	**% Accuracy**

**bbtm vs ntm**	91	94	93.8	91.3	92.5
**bbtm vs ahtm**	88	97	96.7	88.9	92.5

A shows the ability of the program to differentiate between various protein classes without inclusion of evolutionary information or differential amino acid weightings. B shows the improvements given the inclusion of these features. Performance was assessed using the 'leave homologues out' cross-validation.

**Table 4:** Comparison of various predictors with specific examples.

	**BOMP**	**Prof-TMB**	**Pred-TMBB**	**TMB-Hunt: Leave Homs Out**	**TMB-Hunt: Leave One Out**
NalP – Q8GKS5	0^Γ^	12.32	2.92	10.73	10.73
TSX – P22786	1	10.92	2.94	4.47	4.47
FadL – P10384	1	9.47	2.88	0.8	0.8
BtuB – P06129	1	10.39	2.91	10.82	10.82
Secretin – P31700	0^Γ^	3.73^Γ^	2.90	5.48	5.48
Usher – P30130	1	10.46	2.95	10.79	10.79
60 kDA cysteine rich OMP – P26758	0^Γ^	2.42^Γ^	3.03^Γ^	-1.70^Γ^	-1.70^Γ^
Mycobacterial Porin – Q9RLP7	0^Γ^	5.65^Γ^	2.84	7.74	7.74
TolC – P02930	0^Γ^	1.85^Γ^	2.90	6.76	10.64
Alpha hemolysin – O68404	0^Γ^	0.83^Γ^	2.88	9.89	9.89
VDAC – Q60931	1	6.55	2.88	5.24	5.24
Tom40 – Q18090	1	4.79^Γ^	2.92	-1.04^Γ^	-1.04^Γ^
Toc75 – Q43715	0^Γ^	6.50	2.99^Γ^	-1.41^Γ^	1.24
OEP24 – O49929	0^Γ^	3.11^Γ^	2.87	1.55	1.55

All programs were run via their web interfaces, using default settings. Sequences classified as non-bbtm are marked using ^Γ^. BOMP [22] values indicate the number bbtm proteins predicted given the number of sequences queried. Prof-TMB [17] returns a z-score statistic for which 50% of bbtm proteins get a z-score of >= 10 at an accuracy of 80% and 35% bbtm proteins get a z-score >= 6 at an accuracy of 35%. Pred-TMBB [16] returns a threshold score, for which sequences with threshold scores >2.965 are assumed not to be bbtm proteins. Beta-barrel scores, were given for TMB-Hunt. These were calculated without inclusion of evolutionary information, using 'leave homologues out' and 'leave one out' cross-validations. Beta-barrel scores >0 indicate that there is a greater probability that the sequence is from a bbtm protein.

**Table 5:** Proteomes screened.

**Organism**	**Proteins**	**No. signal peptide**	**% proteins with signal peptide**	**No. bbtm protein <E = 1**	**% of proteins with signal peptide bbtm E<= 1**	**% bbtm proteins <E = 1**
Escherichia coli	5341	1032	19.32	87	8.43	1.63
E. coli ^Ш^	4005	782	19.52	69	8.82	1.72
Pseudomonas aeruginosa	5567	1142	20.51	137	12	2.46
P. aeruginosa ^Ш^	5567	1412	25.36	137	9.7	2.46
Staphylococcus aureus	2632	409	15.54	18	4.4	0.68
Aquifex aeolicus	1560	187	11.98	16	8.55	1.02
Chlamydia trachomatis	895	145	16.20	17	11.7	1.89
Thermatoga maritima	1858	265	14.26	12	4.53	0.65
Trepanoma pallidum	1036	203	19.59	12	5.91	1.16
Bacteroides thetaiotaomicron	4778	1614	33.78	131	8.12	2.74
Deinococcus radiodurans	3182	689	21.65	25	3.62	0.76
Rhodopirellula baltica	7325	1584	20.66	49	3.09	0.67
Plasmodium falciparum ^Ш^	9178	1613	17.57	3	0.18	0.03
Arabidopsis thaliana	28860	5569	19.30	23	0.41	0.07
Caenorhabditis elegans ^Ш^	22561	5778	22.60	26	0.45	0.12
Saccharomyces cerevisiae	5866	651	11.09	4	0.61	0.07

Several proteomes were screened, representing the major kingdoms of life. Proteomes were first filtered for sequences with signal peptides. Remaining sequences were then each queried, returning bb scores and E-value statistics. All proteomes were downloaded from the NCBI FTP site except those denoted ^Ш^, downloaded from Uniprot/SwissProt for superior annotation.
